# Optimized planning for intraoperative planar permanent‐seed implant

**DOI:** 10.1120/jacmp.v3i3.2566

**Published:** 2002-06-01

**Authors:** Albert Y. C. Fung, Howard I. Amols, Marco Zaider

**Affiliations:** ^1^ Department of Medical Physics Memorial Sloan‐Kettering Cancer Center 1275 York Avenue New York New York 10021

**Keywords:** optimization intraoperative planar seed implant

## Abstract

We describe a fast, PC‐based optimization planning system for a planar permanent‐seed implant. Sites where this system is applicable include brain, lung, and head and neck. The system described here allowsplacing ribbons of different strengths and of different lengths along and across the implant plane. The program takes full advantage of the availability of different source strengths in inventory, and attempts to find configurations of ribbons that result in optimal dose uniformity over the prescription plane. Dosimetry is based on the AAPM TG 43 Report [R. Nath *et al*., Med. Phys. 22, 209–234 (1995)]. Compared with TG 43 parameters, the classical tables underestimate the I‐125 source strengths needed by 40%. The use of several source strengths improves the plan. Typical optimization yields dose uniformity of 10%, and computing times are within 2–3 min No further enhancement is obtained if ribbons are placed in a grid pattern as opposed to the (simpler) arrangement along parallel lines. Nor is it valuable to have variable ribbon lengths. For an I‐125 implant the optimization system described here is a practical alternative to the (strictly speaking inapplicable) classical systems. It calculates correctly the total source strengths, and ‐ most notably ‐ generates plans with optimal dose uniformity. The fast computing time is well suited for planning during surgery in the operating room.

PACS number(s): 87.53Jw, 87.53.Tf

## INTRODUCTION

The work described in this article stems from the observation that intraoperative permanent implants at sites of residual disease (tumor bed), such as brain, lung, and head and neck, are regularly performed without any treatment plan. A typical case at Memorial Sloan‐Kettering Cancer Center (MSKCC) is the interstitial implantation of skull base tumors: following surgical resection of the tumor, I‐125 seeds, evenly spaced in Vicryl suture (ribbons), are implanted by free hand at a distance of 0.5‐1 cm from each other. The dose prescription is determined by obtaining CT scans of the volume implanted and then selecting an isodose surface that surrounds the region of interest, which is typically determined by the sources themselves.

There exists no good published system for an I‐125 planar permanent implant. Classical implant systems, such as the Manchester, Quimby, or the Anderson nomogram, were originally developed for radium‐equivalent isotopes with high gamma energies. Implants with I‐125 seeds, because of lower energies and greater absorption in tissue, require much higher source strength than those indicated in the classical tables (for the same implant area). There are also important practical constraints for this kind of implant. For instance:

(i) Treatment “planning” must be performed rapidly in the operating room. (ii) The area to be implanted is known only at the time of surgery. (iii) The physicist is not routinely in the position to select the source activity that will optimize the intended dose distribution. (iv) The plan may not be put into operation as designed because of anatomical constraints.

We describe a fast, PC‐based optimization planning system for a planar permanent‐seed implant. In addition, the potential benefit of modifications to the classical implant systems, such as several ribbon strengths, variable ribbon lengths, and crossing ribbons, has never been studied, and these with a computer may significantly improve the quality of the plan while continuing to fulfill the inevitable need for speedy planning. The optimization system described below is meant to produce a maximally uniform dose in a plane a given distance away from the implanted area, in line with the current intent of such treatments.

## METHODS

The optimization system assumes that the implant is rectangular, and that the dose is prescribed in a parallel plane located at a distance *d* above the plane of the sources. Thus, the target volume is a rectangular slab of thickness 2*d*. If the implant is not flat the dose on the concave side will be somewhat larger than that on the convex side. Because it is difficult to measure the implant curvature, at this time it appears impractical to attempt to compensate for its effects.

The program takes as input the isotope type (I‐125 or Ir‐192), a list of source strengths available in the inventory, the length (*L*) and width (*W*) of the implant, the prescription dose, $D_{0}$, and the prescription distance, *d*. The solution describes a pattern of ribbons placed equidistantly (1‐cm apart) along, or perpendicular to the sides of the target volume. It specifies also the source strength used in each ribbon. The program allows one to seek solutions with ribbons along one direction only (*X* or *Y*) and also of variable length. Only solutions that are symmetric with respect to both axes (*X* and *Y*) are considered since the prescription doses are always symmetric.

The set of all possible solutions is limited, and the simplest method of identifying the best solution is to evaluate sequentially all possible ribbon patterns in terms of a cost function. For a more complicated situation, sophisticated techniques such as the genetic algorithm^1^ and simulated annealing^2^ may be used. Specifically, solutions are ranked according to the dose uniformity on the prescription plane. Thus,

(1) $$\text{Uniformity}=\sqrt{\sum_{i}{\left(D_{i}-D_{0}\right)}^{2}}/D_{0}/(\text{number}\;\text{of}\;\text{points}),$$

where $D_{i}$ is the dose delivered at point “*i*”, and the summation is over a predefined configuration of grid points on the prescription plane. As defined, uniformity gives equal score to underdosage and overdosage. Should the physician prefer otherwise, it is very easy to adjust the weights. Dose points are spaced 0.5 cm apart and the gap between seeds is 1 cm; thus, dose points are either just above or in‐between seeds. Since only symmetric solutions are considered, the grid only needs to span one quarter of the area. The total number of dose points is (L and W assumed integers)

(2) $$N_{d}=(L+1)(W+1).$$

In addition to dose uniformity, the program calculates the target coverage (V100, or the percentage of target volume covered by the prescribed dose). The target volume is defined as the slab of tissue bounded by the rectangular plane at distance *d* from the centers of the sources (i.e., the slab thickness is 2*d*). To deduce the target coverage, doses are calculated at points in a three‐dimensional grid of 0.1‐cm spacing inside the target volume. The software also calculates the dose‐volume histogram (DVH) of the plan and isodose distributions in axial planes. Dosimetry is based on the AAPM TG 43 report recommendations.^3^ Full 2D anisotropy functions are employed. The code first calculates, at every grid location, the dose due to ribbons (with unit‐strength seeds) of any feasible length and orthogonal orientation. This dose matrix is calculated before evaluating actual solutions since the dose values in the matrix will be used repeatedly. The computing time is proportional to the number of solutions evaluated. If all ribbon‐arrangement options are turned on, the total number of possible solutions is

(3) $$N_{s}={\left(A+1\right)}^{U+V}U^{V}V^{U},$$

where U=Integer(W/2),V=Integer(L/2), and *A* is the number of available source strengths [the expression A+1 appears in Eq. (3) since zero strength is always permissible for any ribbon]. If solutions are restricted to full ribbon lengths (from end to end of the target area) then the number of solutions is reduced to ${\left(A+1\right)}^{U+V}$. It is obvious that the computing time increases rapidly with the available source strengths and also *L* and *W*. A typical solution is obtained in a few minutes on a Pentium III computer.

## RESULTS AND DISCUSSION

The availability of seeds with several source strengths will obviously improve the plan. This may not be impractical if, given a large number of patients, unused seeds are stocked. At MSKCC, for instance, a standing order of seeds delivered once a month is in effect. New iodine suture seeds have usually air kerma strength of 1.2 U. After one month, this will decay to 70% of its original value (half‐life of 59.4 days). Therefore, it is commonly the case that a series of decreasing source strengths 70% of one another is obtainable.

In the case of a planar implant, an important criterion for plan evaluation and optimization is dose uniformity over the prescription plane. By contrast, in a volume implant, such as the prostate, target coverage and conformity are also invoked. For a planar implant coverage alone (without other constraints) is not sufficient since one can always “heat up” the implant with high activity seeds and achieve 100% coverage, at the expense of conformity and/or excessive dose to the surrounding normal tissue. In the case of brain implant, the normal brain tissue is itself a critical organ to be spared as much as possible. Internal uniformity is important in a volume implant but is misleading in planar implant, since the volumes closest to the radioactive sources are inevitably hot, and such volumes constitute a large fraction of the planar target volume. Uniformity over the prescription plane automatically achieves a compromise between coverage and conformity. It is a single criterion (instead of two) and thus, no extra weighting factor needs to be used.

**Figure 1:**
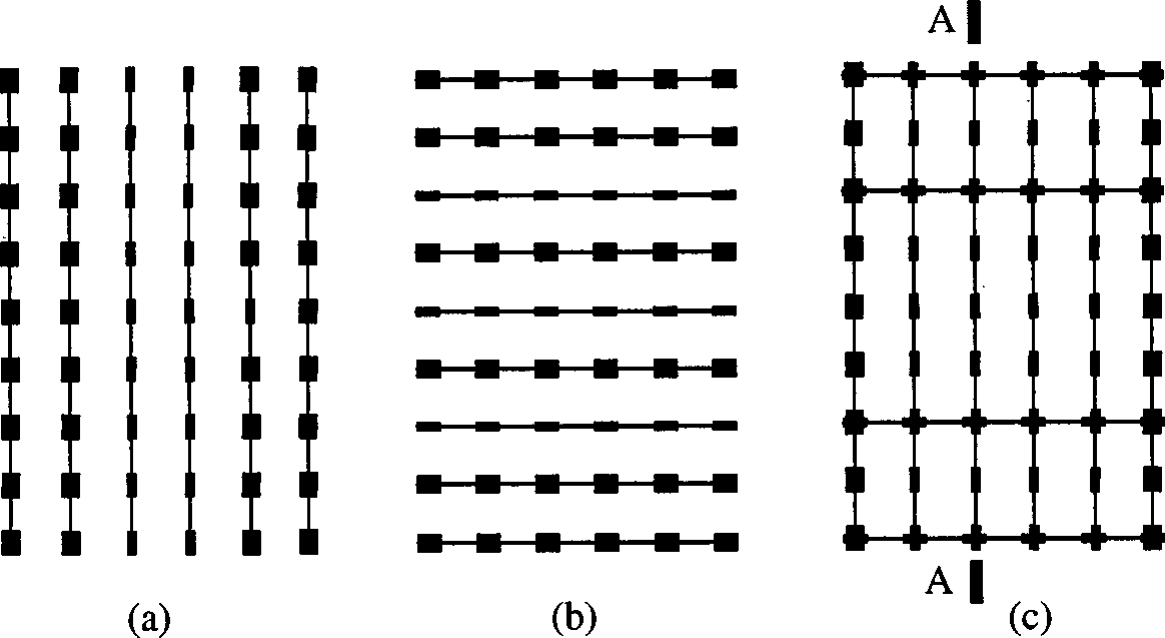
Optimized solutions for an 8×5 cm planar implant, with ribbons along (a) the long direction only, (b) the short direction only, and (c) both orthogonal directions. The seeds shown with larger “diameters” are each of 1 U, and those with smaller “diameters” are of 0.5 U. (In reality, sources have the same diameters regardless of strength.)

**Figure 2:**
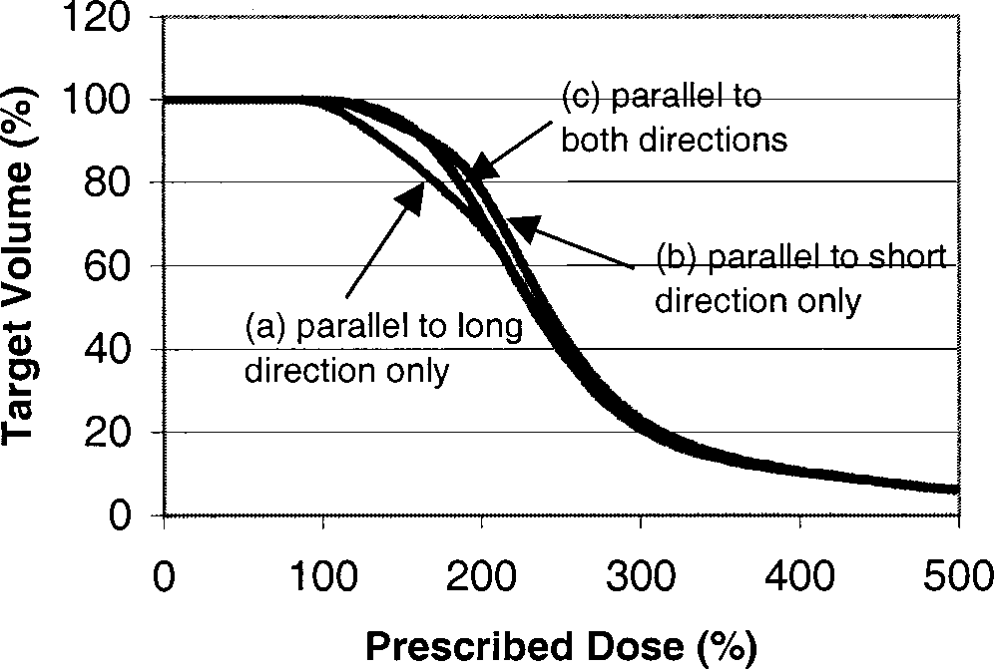
Dose volume histograms of optimized solutions for a 8×5 cm planar implant.

**Figure 3:**
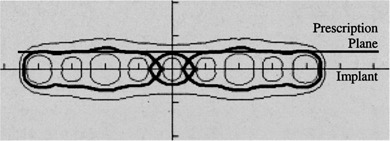
Cross‐sectional plane *A* [indicated in showing the isodose of the optimal plan for an 8×5 cm planar implant. The three isodose curves are 200 Gy, 150 Gy (prescribed dose, thick line), and 110 Gy. The prescription plane is 0.5 cm from the implant plane.

The optimization allows for ribbons in either one of two orthogonal directions. We have also studied the benefit of laying ribbons concurrently in two directions instead of only one direction. It appears that in nearly every case studied, the resulting dose distributions using both directions and either direction are very similar. The example shown in Fig. 1 is for a $8\times 5{\text{cm}}^{2}$ planar implant in which 150 Gy is prescribed at 0.5 cm distance. The available iodine source strengths are 0.5 and 1.0 U. The figure shows the optimized solutions allowing for ribbons parallel to (a) long direction only, (b) short direction only, and (c) both orthogonal directions. The “thick” seeds have 1.0 U while the “thin” ones are of 0.5 U. The seed arrangements of the three solutions are obviously quite different. However, differences between the three solutions (see Table I) are unlikely to have any clinical significance. Figure 2 graphs the corresponding dose volume histograms. Another consideration in using ribbons in both directions is that two seeds at the same spot cannot be placed in exactly the same level, but have to be on top of each other, resulting in slight deviation from the intended dose distribution. Figure 3 is a cross‐sectional plane *A* [indicated in Figure 1(c)] showing the isodose of the optimal plan. Although pockets of cold spots appear within the target volume, the target coverage is in fact 99.93%, i.e., the total volume receiving less than the prescribed dose is only 0.07% of the target volume. The Ir‐192 ribbons we order are of ten‐seed length, with 1‐cm seed separation that gives 9 cm length from end to end. We do not really stock ribbons of various lengths, but we can cut with scissors an original ribbon to any length dictated by the plan. The program does not tract the number of ribbons available. When an activity is put in, it assumes the number of ribbons will be enough. The option of different ribbon lengths, which our program permits, does not seem to be helpful in achieving a more uniform dose distribution. With variable lengths, the computing time for implants larger than $5\times 5{\text{cm}}^{2}$ and with two or more source strengths is prohibitively long (much more than several minutes) and for nearly every optimized plan full‐length ribbons are used anyway. Therefore, we do not turn on this option at all in clinical use.

**Table I:** Comparison of plans allowing for ribbons parallel to (a) the long direction only, (b) the short direction only, and (c) both orthogonal directions.

Ribbons parallel to	Total source strength (U)	Uniformity (%)	Target coverage (%)
Long direction only	4.5	11.6	98.97
Short direction only	4.5	10.4	99.92
Both orthogonal directions	4.8	9.6	99.93

**Figure 4:**
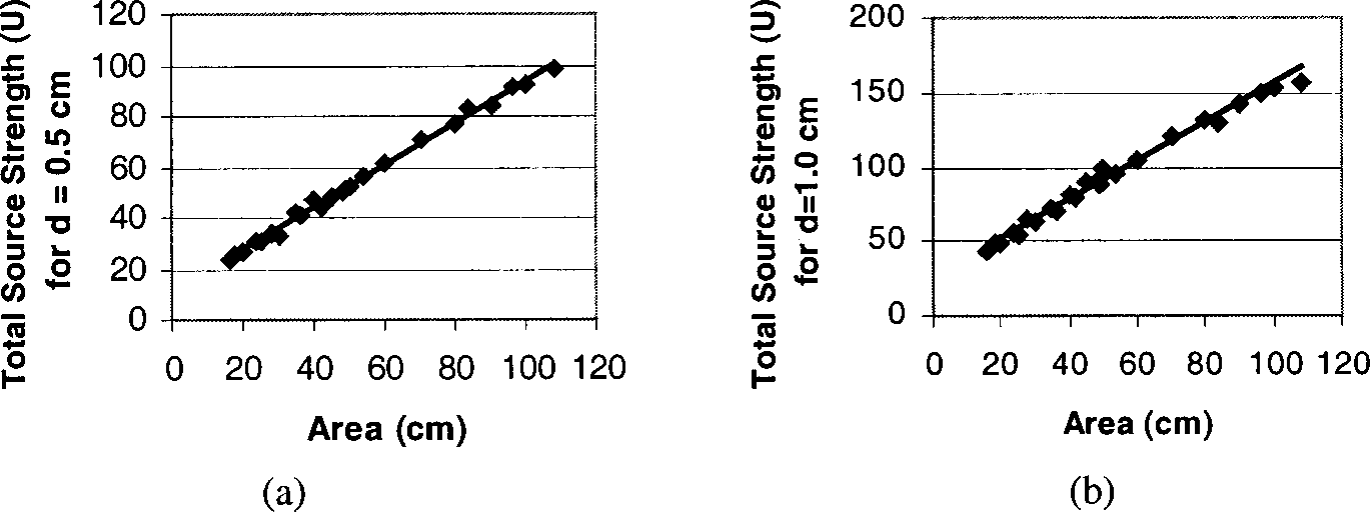
Total source strength (U) for an I‐125 planar permanent implant as a function of area for a 150 Gy dose prescribed at (a) d=0.5 cm and (b) d=1.0 cm.

For hospitals that do not have a standing order, single source strength will probably be ordered for each patient. Our optimization program can still generate the best plan achievable with the limited available source strength. Nevertheless, since orthogonal directions and variable length ribbons seem not worth the trouble, a nomogram or similar formula will suffice for a single strength implant. Although a nomogram for an I‐125 implant was published in the literature,^4^ it was for spheroidal and cylindrical volume only and not for planar volume, and it was derived from the Ir‐192 nomogram and gave inappropriate source activities for I‐125.^5^ We use the optimization software to generate nomogram‐like formulas for hospitals that only have single activity seeds. Specifically, we record the implant areas for 50 patients in the past (to obtain a realistic distribution of treatment areas), and re‐run the plans with the optimizer. All 50 implants were rectangular. The area of the implants ranged from 25 to 108 cm^2^, and the lengths ranges from 3 to 12 cm. The aspect ratio (=length/width) averaged 1.4, with a maximum of 2.0. The results for a prescription dose of 150 Gy are plotted in Fig. 4. The fitted lines for the total source strengths (U) are

(4a) U=0.83×area+10.8   for   150   Gy,   d=0.5  cm,

(4b) U=1.31×area+25.4   for   150   Gy,   d=1.0  cm,

where area is in cm^2^.

The total source strength required is proportional to the prescription dose, therefore the total source strength for any dose can be deduced from these formulas. Strictly speaking, these formulas are good for only for Amersham I‐125 model 6720 suture seeds (same dosimetry as model 6711), and needs to be scaled by a different dose rate constant (cGy/U) for other brands of seeds. We have never treated a patient for distance other than 0.5 or 1.0 cm, hence we have not produced similar formulas for other distances. Note that these fitted lines are based on optimized plans, irrespective of our past actual implant plans, which may or may not be optimal. Among the 50 implants, only one had an aspect ratio of 2. We have run the optimization for aspect ratios as high as 3, and the source strengths from formulas have less than a 10% difference from the optimized values. When the aspect ratio went up to 4, the algorithm in general took too long to finish (more than 10 min). Hence, up to an aspect ratio of 3, the formulas provide valid source strength values, and our program can generate an optimized result in reasonable time.

To use the formulas, first obtain the single individual source strength available, the implant area, and the prescription dose and distance. Calculate the total source strengths needed with the appropriate formula. The total source strengths can be divided by the individual source strength and the number of seeds in one ribbon to obtain the number of ribbons required, and hence the ribbon spacing. As an example, if 0.6 U I‐125 suture seeds are available for a 4×6 cm implant with 120 Gy prescribed at d=0.5 cm, the total source strength required will be (0.83×24+10.8)×120÷150=24.6 U. Since each ribbon has seven seeds (6 cm end‐to‐end), the number of ribbons needed will be 24.6÷0.6÷7=5.85. Hence, one should use six ribbons (to the nearest integer), and the spacing between ribbons will be 4÷(6–1)=0.8 cm.

## CONCLUSION

To summarize, for I‐125 implants the optimization system described here is a practical alternative to the (by and large invalid) classical systems. This is not surprising, since the classical plans are among those being evaluated during optimization. It calculates correctly the total source strengths, takes full advantage of the availability of different source strengths in the inventory and, most importantly, generates plans according to pre‐assigned dose uniformity requirements. Because the computing time is of the order of a few minutes, it is well suited for planning during surgery in the operating room.
